# Effectiveness and implementation of a community-based prevention programme targeting anabolic androgenic steroid use in gyms: study protocol of a quasi-experimental control group study

**DOI:** 10.1186/s13102-016-0062-9

**Published:** 2016-11-17

**Authors:** Yasmina Molero, Johanna Gripenberg, Ann-Sofie Bakshi

**Affiliations:** 1 Department of Clinical Neuroscience, STAD, Centre for Psychiatry Research, Karolinska Institutet, Stockholm, Sweden; 2 Department of Medical Epidemiology and Biostatistics, Karolinska Institutet, Stockholm, Sweden

**Keywords:** Anabolic androgenic steroids, Doping prevention, Nutritional supplements, Polysubstance abuse, Recreational sportspeople, Community-based prevention, Implementation

## Abstract

**Background:**

During the past decades, concerns about increased anabolic androgenic steroid (AAS) use among recreational sportspeople have been raised, yet there is a paucity of AAS prevention efforts targeting this group. Accordingly, doping prevention efforts aimed at gyms have been recommended. The overall objective of the present project is to examine a prevention programme named 100% Pure Hard Training (100% PHT), which targets AAS use among recreational sportspeople training in gyms. Specifically, the project aims to: 1) assess the prevalence of AAS, and its associations with alcohol, illicit drugs, and nutritional supplements use; 2) examine whether 100% PHT can decrease AAS use in gyms, and 3) provide insights into which factors facilitate and/or impede implementation of the programme.

**Methods/design:**

The intervention group consists of 27 gyms, and 27 gyms serve as controls. Intervention gyms take part in 100% PHT, a community-based programme involving several components: (a) training of key stakeholders (i.e., gym staff, gym owners, local police, and municipal prevention coordinators) regarding AAS use; (b) developing an action plan for AAS prevention for each gym; (c) certification of gyms that follow 100% PHT; (d) cooperative relationship between stakeholders; (e) annual follow-up of gyms. The project consists of two studies: Study A will examine the prevalence of AAS use and the effectiveness of 100% PHT (aims 1 and 2), and data for Study A will be collected using questionnaires distributed to gym attendees at two assessment points: baseline (pre-intervention) and follow-up (post-intervention). Study B will evaluate the implementation of 100% PHT (aim 3), and semi-structured interviews with participating stakeholders will be carried out post-intervention.

**Discussion:**

Knowledge gained from the present project can be used to develop community-based doping prevention efforts aimed at recreational sportspeople training in gyms. Furthermore, it can provide insights into which factors are important for successful implementation of AAS prevention programmes that target gyms. Results are also expected to yield information on the prevalence of AAS use as well as associations between the use of AAS and other licit and illicit substances, including nutritional supplements, among recreational sportspeople.

**Trial registration:**

The study was registered retrospectively at isrctn.com (Identifier: ISRCTN11655041; Registration date: 3 November 2016;).

**Electronic supplementary material:**

The online version of this article (doi:10.1186/s13102-016-0062-9) contains supplementary material, which is available to authorized users.

## Background

Anabolic androgenic steroids (AAS) are a class of hormones that can be used to enhance athletic performance and stimulate muscle growth. AAS are classified as illegal or controlled substances in many countries, yet their use has increased during recent years, particularly among non-competitive recreational athletes [1, 2]. The lifetime rate of AAS use is reported to be 3.3% globally, with a higher prevalence among males (6.6%) than females (1.6%) [1]. The lifetime rate of AAS use in Sweden is estimated to be 4.4%, which is above the global average and the highest reported rate in the Nordic countries [3]. Prevalence rates are, however, uncertain due to problems with the reliability and validity of measurements [1].

**Fig. 1:**
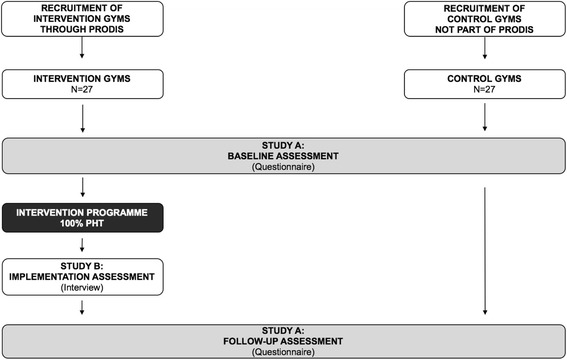
Study design flowchart

**Fig. 2:**
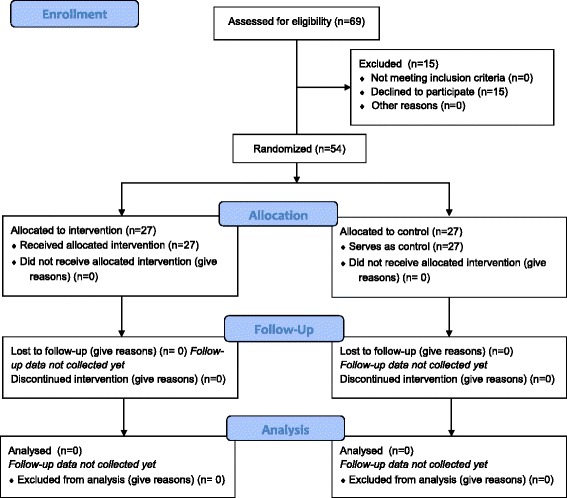
CONSORT 2010 flow diagram

Long-term use of AAS is associated with physical and mental health problems, including hepatic disease, cardiovascular complications, gynecomastia among men, virilization among women, and mood disorders [4–6]. AAS use is also associated with increased aggression and violence [7], although it has been suggested that this association is confounded by concurrent polysubstance use (i.e., the consecutive or simultaneous use of two or more substances) [6, 8].

Traditionally, AAS have mainly been used by professional athletes and bodybuilders. However, AAS use in recreational sports is increasing, and prevalence rates among recreational sportspeople are now believed to have surpassed rates among professional athletes [1], making this a public health problem. In their recent report, *The European Commission Group of Experts on AAS use in recreational sports* concluded that there are few studies on AAS use, and that this compares unfavourably with alcohol and drug research [9]. They have identified three key elements that should be addressed in AAS research to inform policy, practice, and interventions: information on AAS prevalence, use of other substances (beyond AAS), and determinants and correlates of AAS use. Furthermore, they suggest that gyms are important target arenas for preventive efforts [9].

## AAS and the use of other substances

It has been proposed that AAS use may be part of a multi-drug use pattern [10–12]. Studies have shown that people who use AAS are more likely to use alcohol and illicit drugs [10, 11, 13], although the association varies based on the type of drug [11]. AAS use shares several characteristics with misuse of other substances, e.g., withdrawal syndrome, continued use despite adverse effects, and maladaptive behavioural patterns [14]. Moreover, concomitant AAS and drug use is associated with increased risks of mortality, and negative psychophysical effects, possibly due to the combined effects of substances [15]. Consequently, it has been proposed that AAS prevention efforts should also focus on the role of polysubstance use [15].

The use of nutritional supplements is widespread among AAS users [16–19], as such supplements are suggested to improve muscle growth, increase alertness, boost metabolism, and decrease weight or body fat. However, intake of supplements is not unproblematic, as many may be contaminated with stimulants and prohormones (i.e., anabolic steroid precursors) [20–22]. It has been proposed that consumption of nutritional supplements increases the risk of using AAS [17]. By acting as gateway substances, they could lead up to AAS use through gradually increasing involvement in performance-enhancing practices [16, 23]. However, the research is inconclusive, and few studies outside elite sports have examined concomitant use of nutritional supplements and AAS. *The European Commission Group of Experts on AAS use in recreational sports* has identified this as a key barrier to the implementation of AAS prevention programmes, concluding that there is a need for further research to examine the association between nutritional supplements and AAS use [9].

## Settings

The lifetime prevalence of AAS use is consistently higher among gym attendees than in the general population; the reported lifetime AAS use in gym samples ranges between 4% and 24.5%, depending on the type of gym and geographical region [1, 17, 24–26]. Gyms have thus been suggested to contribute to the development of AAS use [27] and to be of importance in studying and preventing AAS use [9, 17]. In recent years, anti-doping efforts have shifted from treatment (i.e., after AAS initiation) to preventive educational strategies [28], and combined educational programmes and practical strength training programmes targeting adolescents and students have shown preventive benefits [29]. It has also been recommended that prevention efforts should involve key stakeholder groups [30]. However, prevention programmes targeting young adults and adults are scarce, and knowledge about their effectiveness is limited, particularly concerning prevention programmes targeting recreational sportspeople.

Since 2008, Prevention of Doping in Sweden (PRODIS), a national anti-doping network involving several key stakeholders engaged in doping prevention in society, has disseminated 100% Pure Hard Training (100% PHT), a community-based programme aimed at reducing doping use among recreational sportspeople training in gyms. 100% PHT has been developed by STAD,^1^ which also coordinates PRODIS. The main intervention components in 100% PHT include: training gym staff in doping prevention, doping policy work, and enforcement and cooperation on doping prevention efforts between key stakeholders (gym staff and gym owners, police, and municipal prevention coordinators). Thus far, approximately 500 gyms in Sweden have participated in the prevention programme. However, the effectiveness and implementation of the programme have not yet been evaluated.

## Objective and research questions

The overall objective of the project is to examine the effectiveness and implementation of the prevention programme 100% PHT, which targets AAS use among recreational sportspeople in gyms. The results are expected to generate information that can be used for doping preventions aimed at recreational sportspeople.

Specifically, the project aims to:

1. Assess the prevalence of AAS, and its associations with alcohol, illicit drugs, and nutritional supplements use among gym attendees.
2. Examine whether a community-based intervention programme targeting gyms can decrease AAS use in these venues.
3. Study the implementation of the intervention programme to provide insights into the factors that facilitate and/or impede implementation.

## Methods/design

### Design

This project consists of two studies (Fig. 1):

Study A measures the prevalence of AAS use among gym attendees (aim 1) and examines the effect of the prevention programme at the gyms where 100% PHT has been implemented (aim 2).

Study B evaluates the process of implementing 100% PHT, focusing on factors that facilitate and/or impede the implementation process (aim 3).

Study A has a quasi-experimental control group design, where 27 gyms are part of an intervention programme, and 27 gyms serve as controls. To study the prevalence of AAS use and the effectiveness of the intervention programme, data will be collected through questionnaires at two assessment points: baseline (pre-intervention) and follow-up (post-intervention). Study B is a qualitative implementation study; data will be collected through semi-structured interviews with key stakeholders taking part in the intervention programme. The interviews will be carried out post-intervention (for the SPIRIT 2013 Checklist, please see Additional file 1).

### Participants

Participants in the current study will include two categories:

- Study A: Approximately 2000 gym attendees have taken part in the pre-intervention assessment, and a minimum of 1600 gym attendees will take part in the post-intervention assessment.
- Study B: Approximately 30 key stakeholders in the intervention group (staff and owners at the intervention gyms, local police and municipal prevention coordinators, gym attendees, current and former users of AAS) will be interviewed about their perspectives on, and experiences of, the intervention programme and doping prevention.

### Procedure

Prevention of Doping in Sweden (PRODIS) is a national network comprising governmental agencies, universities, county administrative boards, municipal prevention coordinators, representatives from the police force and gyms, the Swedish Sports Confederation, and the Anti-doping Hot-line (a national helpline one can call anonymously with questions about doping). The 100% PHT programme is disseminated nationally through PRODIS. PRODIS and 100% PHT were initiated in 2008, and thus far, approximately 500 gyms in Sweden in approximately 100 municipalities (out of the total of 290 municipalities in Sweden) are participating in PRODIS and have implemented the 100% PHT programme. Recruitment of new gyms to the programme is currently underway.

For the quasi-experimental study (Study A), 27 gyms have been selected as intervention gyms, and 27 gyms have been selected as control gyms. Intervention gyms include those gyms that had agreed to participate in 100% PHT during the spring of 2015. Control gyms are gyms not eligible for the intervention programme because they are not located in one of the approximately 100 municipalities (out of the total of 290 municipalities in Sweden) involved in PRODIS.

In the spring of 2015, a pre-intervention assessment was carried out. In this first assessment, a questionnaire was distributed to gym attendees at both intervention gyms and control gyms on a weekday afternoon/evening. The distribution of questionnaires was handled by research staff and not by gym staff. The goal was to collect 30 to 45 questionnaires per gym. If it was not possible to collect the requested number of questionnaires on one occasion, the data collection continued on another occasion close to the original collection day (this was, however, unusual). During the data collection, research staff were placed inside the gym by the entrance, where they invited arriving gym attendees to participate in the study. All gym attendees above age 18 were invited to participate in the study. Gym attendees who agreed to participate were asked to fill in a questionnaire. To guarantee anonymity, participants filled in the questionnaire anonymously and then placed it in an envelope and sealed it, before handing it over to the research staff. In the pre-intervention assessment, 2631 gym attendees were asked to participate in the study. Of those, 1969 gym attendees agreed to participate (996 from intervention gyms, and 973 from control gyms). The response rate was 74.8%.

After the pre-intervention assessment, intervention gyms commenced the prevention programme 100% PHT. Control gyms continued as usual.

By the end of 2016, a post-intervention assessment will be initiated. Again, the same questionnaire will be distributed at both intervention and control gyms in the same manner as in the pre-intervention assessment. Furthermore, semi-structured interviews with key stakeholders will also commence by the end of 2016 to collect data for Study B. The aim of the interviews is to examine implementation of the intervention programme, and to identify factors that have facilitated and/or impeded the implementation process.

Staff specialized in the implementation of public health programmes at the research centre STAD (Stockholm Prevents Alcohol and Drug Problems) are responsible for conducting the intervention programme in all gyms recruited through PRODIS. Further, researchers at STAD are responsible for the data collection, analyses, and interpretation of the current study (Fig. 2).

### Intervention components

The intervention includes the following components:

- Staff and owners at intervention gyms take part in a day-long educational training programme with information on the symptoms and consequences of AAS use, nutritional supplements, doping laws, test methods for detecting AAS use, and techniques for conveying information about AAS to gym attendees. Local police and municipal prevention coordinators also take part in the training. Furthermore, gyms receive AAS information material (posters and brochures) to be distributed at the gyms.
- Each gym develops a written doping action plan and a policy document for AAS prevention, with support from the municipal prevention officer. The policy document and action plan are tailored to the needs of each gym.
- Gyms are certified and receive a diploma. Requirements of certification include educational training of gym staff (required for all staff members that work at least 50%, i.e. 20 h per week.), the production of a policy document and an action plan for AAS prevention, a cooperative relationship with local police and the municipal prevention coordinator, and the appointment of a gym employee responsible for AAS prevention at the gym. The gym is also required to have a folder made available for all staff members, which includes information on AAS use and prevention, contact information to the local police and the municipal prevention coordinator, the long-term action plan, and the policy document.
- Close cooperation between stakeholders is encouraged. This includes follow-ups by the municipal prevention coordinator and regular visits to the gyms by the local police to inform about AAS use and to carry out inspections (in Sweden, AAS are listed as controlled substances, thus personal use, possession and supply are crimes punishable by up to six years in prison).
- The municipal prevention coordinator performs an annual assessment to examine whether the gyms are continuing to meet the requirements of certification (e.g., training new staff members and updating the folder). Gyms that do not fulfil the requirements have six months to address this, or they lose their certification.

### Control components

Gyms in the control group continue as usual between baseline and follow-up assessments.

### Measures

The data in the current study consist of both questionnaire responses (Study A) and semi-structured interviews (Study B).

Study A: To assess the use of AAS, alcohol, illicit drugs, and nutritional supplements in gyms at baseline (i.e. pre-intervention), a questionnaire was distributed to gym attendees at both intervention and control gyms. The questionnaire consists of 25 questions, and includes questions on sociodemographic variables (i.e., occupation, and education), training frequency, AAS, alcohol and drug use, use of nutritional supplements, offers and acquisition of AAS, and attitudes towards AAS use, doping prevention, and AAS regulations. At the follow-up assessment (post-intervention), the same questionnaire will be distributed to gym attendees at both intervention and control gyms.

Study B: To provide insights into the factors that facilitate and/or impede implementation of the intervention programme, semi-structured interviews with key stakeholders (i.e. local police, municipal prevention coordinators, gym staff, gym attendees, users and former users of AAS) will be carried out. The interviews will focus on specific arenas of the implementation: the cooperation between stakeholders, integration of the intervention programme 100% PHT at the gyms, whether the programme is perceived as an effective method for AAS intervention, factors that facilitate and/or impede the implementation process, and long-term maintenance of the intervention programme. Interviews will last 60–90 min, will be recorded digitally, and transcribed verbatim. Informants will be anonymous.

### Statistical analysis

For the pre- and post-intervention assessments in the quasi-experimental study (Study A), the characteristics of participants at intervention and control gyms will be presented. For continuous measures, means and standard deviations will be calculated. For categorical measures, percentages and frequencies will be presented. The effect of the prevention programme on AAS use will be evaluated using effect size estimates. For all analyses, 95% confidence intervals will be applied.

### Qualitative analysis

Analysis of the interview data (Study B) will be made using thematic content analysis, as described by Braun and Clarke [31]. The interview transcripts will be read repeatedly to identify categories of relevance to the research aims and questions. The emerging categories will then be grouped according to coherence in topic, as well as in relation to the research aims and questions. Thereafter, themes will be constructed. The themes will therefore consist of topics that reoccur throughout the interview dataset and that are relevant to the research aims.

### Sample size

The current study consists of cross-sectional data collected at three points in time: pre-intervention assessment (questionnaire), post-intervention assessment (questionnaire), and post-intervention implementation assessment (semi-structured interviews).

Study A: Previous studies have shown that the effect of prevention programmes is rather low, typically about 15-20% [32, 33]. To measure the intervention effect, a minimum of 40 gyms (20 intervention gyms, and 20 control gyms) and a minimum of 1600 participants (800 individuals per condition) will be required at each data collection point to achieve a power of 80% at an alpha level of .05 (2-tailed). In the current study, 27 intervention gyms and 27 control gyms have been enrolled. In the pre-intervention assessment, a total of 1969 individuals completed the questionnaire.

Study B: Participants in the interview study will be selected using purposive sampling, where representatives for all participant groups in the intervention group (i.e., local police, municipal prevention coordinators and gym staff) will be asked to participate. Additionally, gym attendees in the intervention gyms as well as current and former users of AAS will be interviewed. Approximately 30 informants from the key stakeholder groups will be interviewed regarding implementation of the intervention programme.

### Ethical considerations

Participants in all assessments will receive oral and written information about the aims, procedures, and confidential nature of the study. Furthermore, they will be informed that they have the right to ask any questions they wish and to withdraw from the study at any time. In the written information, participants will receive information allowing them to contact the study’s project leader should they have further questions about the study or their participation.

Participation in the quasi-experimental study (Study A) is anonymous; participants will place their questionnaire in an envelope and seal it before handing it over to the research staff. Participants will provide their oral informed consent for the study. Written informed consent will not be collected, thus ensuring that study participation is anonymous. Furthermore, all gyms will be de-identified and the results will be presented for the whole study population (i.e. not on individual gym level).

In the implementation study (Study B), participants are anonymous and pseudonyms will be used. No data derived from the interviews that could potentially lead to identification of the informants will be published. The informants will also be informed, orally as well as in writing, about their right to discontinue their participation in the study at any time during the study process.

## Discussion

AAS use among recreational sportspeople has increased, yet there is a paucity of AAS prevention efforts targeting this public health problem [9]. Accordingly, *the European Commission Group of Experts on AAS use in recreational sports* has recommended prevention efforts that target gyms. Results from the present study will be important, as they can be used to prevent and reduce AAS use in gyms, and also provide insights into the factors that are important for successful implementation of prevention programmes. Knowledge gained from the study will not only be important for evaluation of the large preventive effort across gyms in Sweden, but can also be used internationally to develop community-based doping prevention strategies targeting recreational sportspeople. We also hope to add to the literature on the association between the use of AAS and of other licit and illicit substances, including nutritional supplements. These supplements in particular have been identified by *the European Commission Group of Experts on AAS use in recreational sports* as an arena that needs further research [9].

The present study has limitations that may affect the interpretation of future results. The intervention is a multi-component programme, and as such it may prove difficult to determine which part of the programme is the most effective for AAS prevention (e.g., staff training or stakeholder mobilization). On the other hand, community-based interventions that combine several components have proven to be successful in reducing use and misuse of alcohol and illicit drugs [34, 35], and it has been suggested that it is the combination of components that may achieve the greatest effect [34]. Furthermore, the design of Study A is quasi-experimental. Consequently, potential post-intervention reductions in AAS use may not be reflective of actual reductions among gym attendees, but rather reflect a situation in which individuals who currently use or wish to begin using AAS transition to gyms that do not apply doping prevention programmes. Also, rates of AAS use may be underestimated due to social desirability, the illegality of AAS substances, or fear that reported AAS use may attract unwanted attention to the gym.

Important strengths of the study include the large sample size and the allocation of gyms (albeit not randomly) to two conditions: intervention or control. Another strength of the study is that this is, to the best of our knowledge, the first evaluation of a multi-component community-based AAS prevention programme targeting gyms, and the first study to examine both the effect of the intervention as well as the implementation process, thus providing insights into the feasibility and perceived effectiveness of the programme.

To conclude, increasing AAS use among recreational sportspeople is becoming a problem of concern. The current study can improve our knowledge base on efforts to prevent and reduce AAS use among recreational sportspeople training in gyms, and the results can be used to further develop AAS prevention programmes.

## Additional file

Additional file 1: The Standard Protocol Items: Recommendations for Intervention Trials SPIRIT 2013 Checklist. (DOC 123 kb)

## Abbreviations

100% PHT: 100% Pure Hard Training
AAS: Anabolic Androgenic Steroids
PRODIS: Prevention of Doping in Sweden
STAD: Stockholm Prevents Alcohol and Drug Problems

## Footnotes

1 STAD (Stockholm Prevents Alcohol and Drug Problems) is a research and development centre; its purpose is to identify, develop, implement and evaluate methods for community-based alcohol and drug prevention. STAD is a unit within the Centre for Psychiatry Research, a joint operation between Stockholm County Council and Karolinska Institutet.
